# Nontuberculous Mycobacterial Disease and Molybdenum in Colorado Watersheds

**DOI:** 10.3390/ijerph17113854

**Published:** 2020-05-29

**Authors:** Ettie M. Lipner, Joshua French, Carleton R. Bern, Katherine Walton-Day, David Knox, Michael Strong, D. Rebecca Prevots, James L. Crooks

**Affiliations:** 1 National Jewish Health, Denver, CO 80206, USA; strongm@njhealth.org (M.S.); crooksj@njhealth.org (J.L.C.); 2 Department of Epidemiology, Colorado School of Public Health, Aurora, CO 80045, USA; 3 Department of Mathematical and Statistical Sciences, University of Colorado Denver, Denver, CO 80204, USA; joshua.french@ucdenver.edu; 4 U.S. Geological Survey, Colorado Water Science Center, Denver, CO 80225, USA; cbern@usgs.gov (C.R.B.); kwaltond@usgs.gov (K.W.-D.); 5 Department of Computer Science, University of Colorado-Boulder, Boulder, CO 80309, USA; david.knox@colorado.edu; 6 National Institute of Allergy and Infectious Diseases, National Institutes of Health, Bethesda, MD 20814, USA; rprevots@niaid.nih.gov

**Keywords:** nontuberculous mycobacteria, watersheds, molybdenum, spatial, Poisson, source water

## Abstract

Nontuberculous mycobacteria (NTM) are environmental bacteria that may cause chronic lung disease. Environmental factors that favor NTM growth likely increase the risk of NTM exposure within specific environments. We aimed to identify water-quality constituents (Al, As, Cd, Ca, Cu, Fe, Pb, Mg, Mn, Mo, Ni, K, Se, Na, Zn, and pH) associated with NTM disease across Colorado watersheds. We conducted a geospatial, ecological study, associating data from patients with NTM disease treated at National Jewish Health and water-quality data from the Water Quality Portal. Water-quality constituents associated with disease risk were identified using generalized linear models with Poisson-distributed discrete responses. We observed a highly robust association between molybdenum (Mo) in the source water and disease risk. For every 1- unit increase in the log concentration of molybdenum in the source water, disease risk increased by 17.0%. We also observed a statistically significant association between calcium (Ca) in the source water and disease risk. The risk of NTM varied by watershed and was associated with watershed-specific water-quality constituents. These findings may inform mitigation strategies to decrease the overall risk of exposure.

## 1. Introduction

Nontuberculous mycobacteria (NTM) are environmental organisms and opportunistic pathogens responsible for an increasingly high burden of lung disease in North America, and indeed worldwide [1,2]. More than 190 NTM species have been identified to date [3]; they have been isolated from a variety of natural environmental reservoirs, primarily soil and water. Environmental conditions related to soil properties, natural source water, and the characteristics of engineered water systems, including the biofilms that form in hospital and municipal water supplies, are believed to contribute to increased concentrations of NTM, leading to greater potential for NTM exposure [4]. Although exposure to NTM is extremely common and the NTM disease is rare, distinct geographic variability of disease has been demonstrated in both general and high-risk populations [5,6,7,8,9,10,11]. Hawaii, Florida and California have consistently shown high disease prevalence [8,10,11]. These geographic differences are not explained by host-related factors, but rather are due to variation in regional environmental conditions. Specific soil and water-related factors that favor NTM growth and persistence likely increase the risk of NTM exposure within given environments. Previous epidemiologic studies [6,8,11,12,13,14,15] demonstrate that specific environmental factors may interact to create conditions favorable for increased concentrations of NTM organisms, thereby increasing the individual exposure risk in a given environment. However, large gaps remain in our understanding of the geographic variability of NTM.

Identifying determinants of the regional ecology and environmental sources of NTM is of major public health importance [5,10]. The rapidly aging U.S. population has greater risk for developing NTM disease. Explaining the increasing prevalence trends is critical, as NTM patients undergo lengthy and complex treatment regimens, and are often re-infected following initial cure. The lack of evidence-based guidance on environmental risk factors is a critical public health data gap for at-risk populations. 

In our previous study [14], we demonstrated an increased risk of NTM disease within specific watersheds in Colorado. To further explore these findings, we sought to examine why we observed higher disease risk in these areas. The aim of this study was to assess whether watershed water-quality constituents are associated with increased risk of NTM disease in Colorado. We used an ecological design with water-quality data collected or hosted by the U.S. Geological Survey, U.S. Environmental Protection Agency and National Water Quality Monitoring Council with NTM data from patients residing in the State of Colorado and treated at National Jewish Health (NJH), a leading respiratory hospital in Denver. 

## 2. Methods

### 2.1. Data Collection

**Figure 1:**
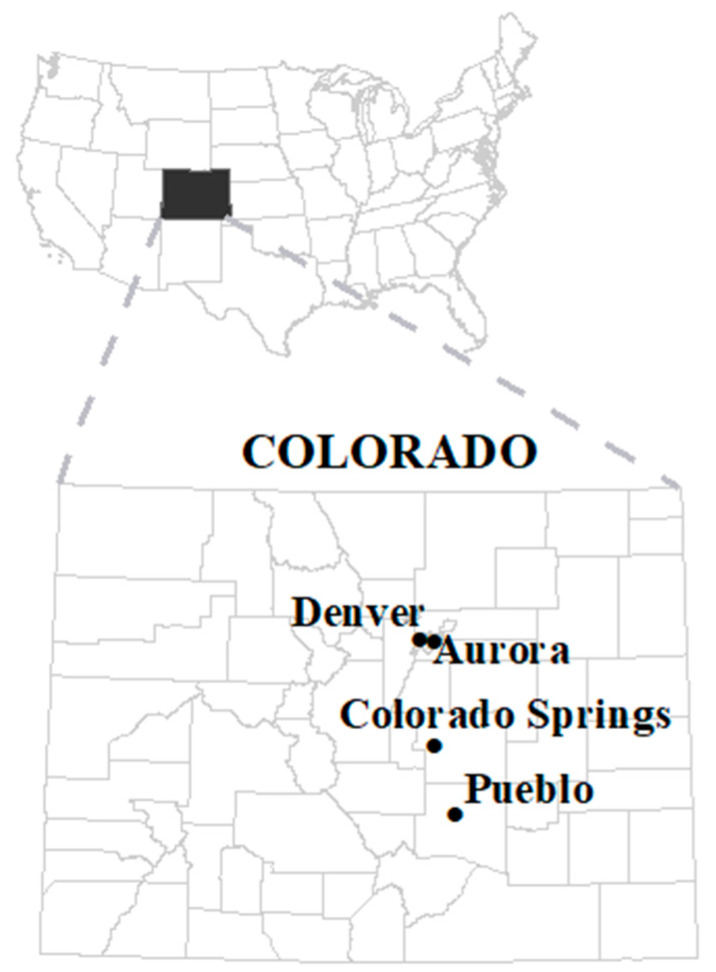
Location of Colorado within the continental United States.

Patient data were obtained from the NJH Electronic Medical Record database. The study population was comprised of all patients with a diagnosis of NTM treated at NJH and who were resident in Colorado (Figure 1) during the study period, from February 2008 through January 2018. Patient address, NTM species, and patient demographic information were extracted from the database. Body site isolation data were not available, therefore NTM disease included pulmonary and extra-pulmonary. This study was approved by the NJH Institutional Review Board (HS-3148). Figure 1 shows the location of the state of Colorado within the continental United States.

#### 2.1.1. NTM Species

NTM species from patient isolates are listed in Supplementary Table S1. 

#### 2.1.2. Socio-Demographic Data

Gridded population density datasets of total population as well as age and racial/ethnic categories were obtained for Colorado during 2010 from the Socio-economic Data and Applications Center (SEDAC) by the Center for International Earth Science Information Network (CIESIN), Columbia University [16]. This dataset contains the following racial/ethnic categories: White Alone, Black or African American Alone, Asian Alone, American Indian and Alaska Native Alone, Native Hawaiian and Other Pacific Islander Alone, Some Other Race Alone, and Two Or More Races. Because Colorado has a majority white population, for this analysis we created two categories: “White Alone” versus all other race/ethnic groups, termed “Non-White”. Population density, age and racial/ethnic categories per watershed were calculated using ArcGIS 10.2 (ESRI, Boston, MA, USA).

#### 2.1.3. Environmental Exposure Data

Watershed boundaries were obtained from the Watershed Boundary Dataset [17] and have been described previously [14]. In this analysis, we used the Hydrologic Unit Code (HUC)-10 watershed level.

#### 2.1.4. Water-Quality Data Compilation

We obtained water-quality constituent data from the Water Quality Portal (WQP) [18], a water-quality database sponsored by the U.S. Geological Survey (USGS), the U.S. Environmental Protection Agency (EPA) and the National Water Quality Monitoring Council (NWQMC). We accessed the WQP on 8 August 2019 and extracted surface-water data. The cleaned dataset used in this analysis required correcting identified unit errors, incorrect latitude or longitude for specific sampling locations, as well as identifying and excluding particular locations that were not surface-water sampling sites. Appendix S1 details the data cleaning steps that were taken to go from the raw data to the cleaned dataset used in the analysis. The cleaned dataset included 62 water-quality constituents from 1,110,882 total samples collected from 7171 unique sampling sites in Colorado during 1 January 2000 through 31 December 2018. We examined the filtered (dissolved) water- sample fractions (filtered indicates that water was passed through a 0.45 -micrometer filter [19]) (Supplementary Table S2). Table 1 presents the median and standard deviation values of the water-quality constituents obtained from the WQP that were used in our analyses.

### 2.2. Statistical Analysis

Analysis of data was performed using the R packages, “rgdal” [20], “sp” [21], “arm” [22], and “dplyr” [23]. All surface-water sampling sites were aggregated by watershed using the R package, “sp”, and the median value of each water-quality constituent was calculated for each watershed using the R package, “dplyr”. The R source code that we created to calculate the watershed medians is available in the Supplementary Materials. Apparent concentration-unit reporting errors were corrected (for example, three orders of magnitude deviations were multiplied by 1000 to align them with the range of the remaining source-specific data). Water-quality constituents were eliminated if data were not available for more than 50 percent of the target watersheds. Following these curation steps, 16 water-quality constituents remained for analysis. We used a natural log transformation of all watershed median variables with a highest to lowest value ratio greater than 3 across watersheds (16 variables). For watersheds with missing data (i.e., watersheds with no data collected for a particular constituent), we imputed the median value using the “imputePCA” function in the “missMDA” R package (version 1.14) [24]. We excluded watersheds in which there were no water samples for any of the water-quality constituents. As a result, we conducted our analyses on 417 out of 575 HUC-10 level watersheds. Drive times between watershed centroids and NJH were calculated using the R “rgeos” package [25]. We categorized watersheds based on whether their centroid center was within a 2.0-h drive to NJH. To account for multiple comparisons, we applied the Bonferroni method to assess statistical significance (*p*-value = 0.007).

#### 2.2.1. Principal Component Analysis (PCA)

PCA was performed on the HUC-10 level dataset (after data were log transformed and imputed) including 16 water-quality constituents using the “PCA” function in the R package, “FactoMineR” (version 1.42) [26]. We retained the top three principal components, which explained 64.6% of the data variability, for further analysis (Supplementary Table S3).

#### 2.2.2. Poisson Regression Models

Five Poisson regression models were constructed to model disease risk as a function of water-quality constituents. Our models used the standard log link function and included the log of the population density within each watershed for the state of Colorado as an offset term to account for the differing population densities in each region. NTM case counts were aggregated by watershed. Age, race/ethnicity, and drive time variables were included in all models to control for potential confounding associated with a hospitable-based population. Relative risk, 95% confidence interval (CI), and a *p*-value are presented for each model variable. (*p* < 0.05). To create Figures 3 & 4, we used the best-fit estimates of the watershed-specific risks from the Poisson models. 

##### Principal Component Regression Model

The first Poisson regression model included the top three principal components from the PCA (Table 2; Model 1). Using the analysis of deviance test, the model containing the top three components demonstrated the best improvement in fit. 

##### Poisson Regression Models with Individual Metals 

From Supplementary Table S3, we identified the individual metals that contributed more than 3% to each statistically significant principal component (components 1 and 3). Principal component 2 was not statistically significant and was therefore not further explored in this analysis. The identified metals from principal components 1 and 3 were added as predictor variables to Poisson Models 2 and 4. We then constructed separate single-exposure regression models (Models 3 and 5) for the metals that demonstrated statistical significance from Models 2 and 4 (*p* < 0.05). 

##### Sensitivity Analyses

We performed two sensitivity analyses of our final results. To investigate how our estimates changed when we relaxed our distributional modeling assumption, we used a negative binomial response distribution instead of Poisson (Supplementary Table S5).

## 3. Results

Our study population comprised 821 patients with NTM disease who had sought treatment at NJH between February 2008 and January 2018 and reside in Colorado. For all NTM patients, the mean age was 64.8 years (±18.1) and the majority of patients (74.4%) were white. Eight hundred and seven patients were available for analysis after accounting for dropped watersheds.

### 3.1. Principal Component Regression Model

Our findings showed that principal components 1 and 3 were significantly associated with disease risk (Table 2; Model 1). 

For every 1- unit increase in source water constituents contributing to principal component 1, there was a 5.4% increase in disease risk (Table 2; Model 1); the highest contributing water-quality constituents included arsenic, calcium, magnesium, molybdenum, potassium, selenium, sodium, and pH (Supplementary Table S3). For every 1- unit increase in source water constituents contributing to principal component 3, there was an 8.3% increase in disease risk; the highest contributing water-quality constituents included aluminum, arsenic, cadmium, manganese, molybdenum, selenium, zinc, and pH. 

The fraction of the population from non-white racial/ethnic groups was a significant protective factor against NTM disease risk. Socio-economic status could be confounding this association because up-to-date gridded socio-economic data were not available for inclusion.

**Figure 2:**
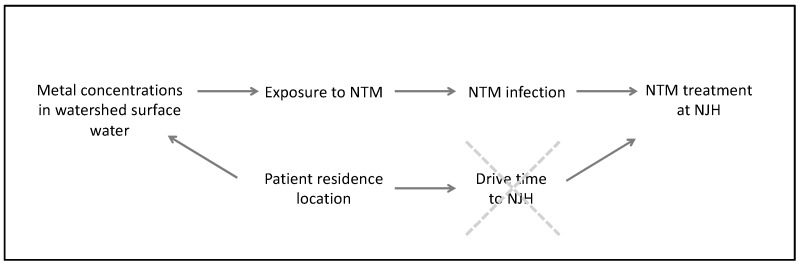
Directed acyclic graph to explore how “drive time” influences disease risk in our study. Controlling for drive time blocks the non-causal backdoor path between metal concentrations in watershed of residence and NTM treatment at NJH.

We created a “drive- time” variable to control for oversampling of patients residing in Front Range communities, where NTM patients are more likely to be seen at NJH than patients outside of this metropolitan area. By accounting for drive time in our models, we block a non-causal backdoor path in the causal diagram (Figure 2).

### 3.2. Poisson Regression Models with Individual Metals 

We modeled the risk of NTM disease as a function of the variables that contributed at least 3% to principal components 1 and 3 (Models 2–5). This threshold captured 50% of the water-quality constituents that were contributing to each component. We examined the variance-inflation factors for principal component 1 and found that magnesium, calcium, potassium, and sodium were highly correlated. In principal component 3, we detected no collinearity. From principal component 1, we observed a significant association between calcium, magnesium, molybdenum and disease risk, while controlling for the presence of the other water-quality constituents, drive- time, age and race (Table 3; Model 2). 

We then modeled the risk of NTM disease as a function of each significant metal from Model 2 in a separate single-exposure model (Table 4; Model 3).

From principal component 3, we observed a significant association between molybdenum and disease risk and a less significant association between arsenic and disease risk, while controlling for the presence of the other water-quality constituents, drive- time, age, and race (Table 5; Model 4).

We modeled the risk of NTM disease as a function of each significant metal from Model 4 in a separate single-exposure model (Table 6; Model 5). 

Molybdenum and calcium remained significantly associated with disease risk (Table 4; Model 3), while magnesium (Table 4; Model 3) and arsenic did not (Table 6; Model 5). Our results indicate that for every 1-log unit increase in molybdenum concentration in the source water at the HUC-10 watershed level, the risk of NTM disease increased by 17% (Table 4; Model 3 and Table 6; Model 5). Our results also indicate that for every 1-log unit increase in calcium concentration in the source water at the HUC-10 watershed level, the risk of NTM disease increased by 19% (Table 4; Model 3). The effect of calcium and molybdenum on disease risk remained statistically significant after controlling for multiple comparisons using the Bonferroni method (7 models; new *p*-value = 0.007). Supplementary Table S4 shows the correlation matrix for the metals tested in Models 2 and 4 (Table 3; Table 5). 

**Figure 3:**
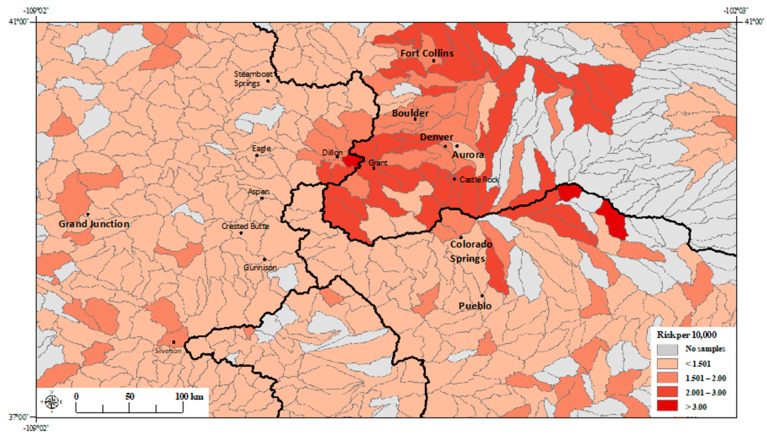
Fitted NTM disease risk estimates per watershed (HUC-10) based on PCA regression model (including principal components 1, 2 and 3) (Model 1; Table 2). *Black lines* represent watershed boundaries of four major watersheds (HUC-2) in Colorado. *Black line* separating the leftmost watershed from the remaining three watersheds represents the Continental Divide. These four major watersheds are divided into 575 HUC-10 level watersheds (boundaries delineated by gray lines). City names are printed in boldface type, town names are printed in smaller font.

**Figure 4:**
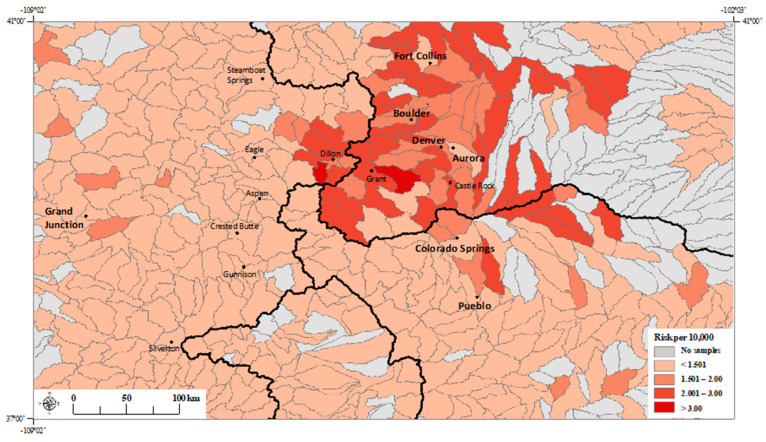
Fitted NTM disease risk estimates per watershed (HUC-10) based on molybdenum regression model (Model 3; Table 4). *Black lines* represent watershed boundaries of four major watersheds (HUC-2) in Colorado. *Black line* separating the leftmost watershed from the remaining three watersheds represents the Continental Divide. These four major watersheds are divided into 575 HUC-10 level watersheds (boundaries delineated by gray lines). City names are printed in boldface type, town names are printed in smaller font.

The estimated risk estimates across Colorado watersheds based on the regression model with the three principal components (Table 2; Model 1), and the regression model with molybdenum alone (Table 4; Model 3 and Table 6; Model 5) are shown in Figure 3; Figure 4, respectively.

We conducted a sensitivity analysis switching the Poisson response to a negative binomial response (Supplementary Table S5). Using this distribution, we ran separate single-exposure models for each of our final exposures: calcium and molybdenum. The estimated coefficients for calcium and molybdenum remained positive and statistically significant (*p* = 0.028 and 0.024, respectively).

## 4. Discussion

We found that the presence of calcium and molybdenum in the source water is associated with an increased risk of NTM disease (Table 3, Table 4, Table 5 and Table 6; Models 2–5). After removing the non-significant metals from the model, we found that for every one-log unit increase in the calcium and molybdenum concentrations in the source water, a 19% and 17% increase in NTM disease risk was observed, respectively (Table 4; Model 3). From our fitted estimates, we observe numerous high-risk watersheds in the mountainous regions to the west of the Continental Divide and along the Front Range to the east of the Continental Divide (Figure 3; Figure 4). Watersheds in the mountainous regions provide most of the water supply to highly populated communities in the Front Range [27]. Molybdenum is also highly abundant in the mountainous regions of Colorado [28].

The effect of molybdenum on mycobacteria has been described previously [29,30,31]. Several molybdenum enzymes in mycobacteria exert important physiological functions. *Mycobacterium tuberculosis* as well as the nontuberculous mycobacteria contain many proteins for the import and utilization of molybdenum, including the molybdate transport proteins modA, modB, and modC, and the molybdenum cofactor biosynthesis proteins moaA, moaB, moaC, moaD, and moaE. Some mycobacteria, including *M. tuberculosis*, contain additional paralogs of the molybdenum cofactor biosynthesis proteins [29]. Molybdenum has been shown to be essential for nitrate assimilation in mycobacteria [30] and is an essential component of many bacterial enzymes involved in carbon, nitrogen, and sulfur metabolism [30]. In *M. tuberculosis*, molybdenum cofactor biosynthesis proteins have been suggested to be associated with pathogenesis [31] and with hypoxic persistence [30] potentially contributing to the ability to shift nitrogen respiration under the oxygen-limiting concentrations that may occur in lung granulomas. This literature suggests a physiological connection linking molybdenum and essential metabolism, potentially affecting pathogenesis and persistence of *M. tuberculosis*. Although this mechanism has not been established for NTM, it offers biological plausibility because NTM and *M. tuberculosis* are phylogenetically related organisms [4].

Our study opens many avenues of research to investigate the influence of molybdenum on NTM growth in water sources as well as in the human host. In a recent Korean study, Oh et al. [32] reported that trace element status is associated with mycobacterial lung disease. The authors demonstrated that patients with pulmonary NTM had higher median molybdenum concentrations in their serum (1.70 μg/L) compared with healthy controls (0.96 μg/L) and patients with pulmonary tuberculosis (0.67 μg/L). Patients and clinicians alike would benefit from knowing whether molybdenum intake from water consumption or from certain dietary profiles (e.g., vitamin supplementation containing molybdenum) increases the risk of infection and/or progression of disease.

Molybdenum is mainly used as an alloying agent in the production of steel because of its strength and ability to withstand high temperatures. Small quantities of molybdenum are essential to human, animal and plant life, and it is present in trace quantities in rocks, soil and water, often at concentrations less than 10 µg/L [33]. The environmental concentrations of molybdenum can vary widely, and in places where molybdenum is processed, the concentrations in soil and water may increase considerably [34]. Molybdenum has “relatively high geochemical mobility—a tendency to enter into solution in water under normal Earth-surface conditions” [34,35]; we hypothesize that perhaps even small amounts of water-soluble molybdenum may act as a metabolic source for NTM in the water supply. Soil moisture is known to influence molybdenum availability: poorly drained wet soils (for example, peat marshes, swampy organic rich soils) tend to accumulate molybdenum to high levels [36]. Likewise, Falkinham and colleagues have repeatedly shown that peat rich soils and brackish marshes are rich in NTM [4,37,38]. In addition, molybdenum can form complexes with organic matter, particularly humic and fulvic acids [39]. Falkinham and colleagues have also reported that humic and fulvic acids support high numbers of *Mycobacterium avium* complex (MAC) species [4,40]. We found that the median value of molybdenum across Colorado watersheds was 4.3 µg/L (Table 1), but reached 325 µg/L at one specific watershed.

**Figure 5:**
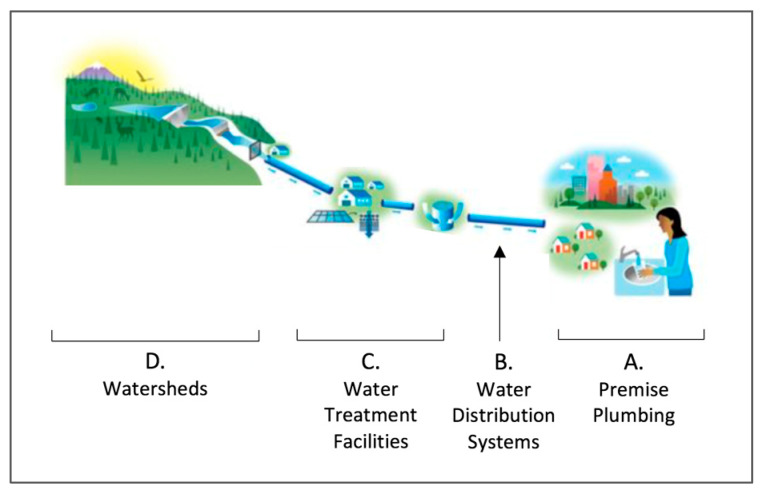
Locations of potential exposure to NTM.

Many studies have examined mycobacterial distributions and abundance in different geographic areas. These studies range from examining the presence of NTM in premise plumbing [6,41,42,43], in the water distribution systems [44], in the water treatment facilities [45] and in the watershed untreated source water [46] (Figure 5) [47].

Studies have demonstrated that NTM exposure and infection occurs in the home (Figure 5A) [42,48,49], with household water as a source of exposure. Lande et al. [41], for example, showed genotypic matches between *M. avium* respiratory isolates and isolates from household plumbing. Studies have shown the proliferation of NTM in water distribution systems upstream from premise plumbing [44] (Figure 5B). However, the entry point for these organisms into the water distribution system and premise plumbing remains unknown. Farther upstream, NTM have been found in water treatment facilities (Figure 5C). King et al. [45] conducted a survey to obtain information on mycobacteria (as well as other microbial pathogens) in source and treated drinking water collected from drinking water treatment plants (DWTPs) across the United States (Figure 5C,D). *M. avium* and *Mycobacterium intracellulare* were detected in 6 out of 24 source water samples and both samples were detected simultaneously at 4 DWTPs. King et al. also identified 10 out of 24 DWTPs that had no mycobacteria detected in source or treated water. The literature indicates that the high-risk and low-risk regions for mycobacterial exposure likely correspond to high and low risk areas for disease.

Although we did not find literature that explain the association of calcium in source water on NTM disease risk, we speculate that for this Colorado dataset the association may be explained in part by the correlation of calcium with molybdenum because of environmental factors, rather than any meaningful link between calcium and NTM bacteria or NTM disease prevalence. Watersheds with greater disease risk and dissolved molybdenum concentrations are more prevalent on the eastern plains of Colorado (Figure 4). Compared to much of Colorado, waters on the eastern plains have generally greater concentrations of major constituents, like calcium, because of evapotranspiration in a warmer and drier climate, and more prevalent sedimentary rocks that weather to release calcium [50]. An association of such rocks with molybdenum release remains to be explored and warrants further research.

Additional research is required to confirm the causal pathway between molybdenum, NTM abundance, and disease prevalence. If molybdenum is a metabolic factor for NTM in the environment, it is plausible that the mycobacteria also utilize this trace metal to survive in the host, a possibility that may explain reports of higher blood-serum molybdenum concentrations in NTM patients [32]. Identification of these factors is critical to develop prevention strategies for minimizing exposure and infection in high-risk regions.

## 5. Limitations

The water-quality data that we extracted from the WQP have their own implicit biases. The sampling locations were not from random or systematically representative locations, rather they were targeted for varying purposes. Although some sites may have been sampled monthly for years, others were sampled only once for a specific project and some watersheds were not sampled at all. Additionally, data were imputed to some watersheds with missing information. Therefore, we cannot predict how much bias may be influencing the resulting median concentrations at each watershed. Additionally, a lack of data on NTM abundance prevents us from correlating high NTM densities in the source water with high prevalence of disease. There are also limitations inherent to our study population, which has been previously discussed [14]. We included a drive- time variable to control for oversampling of patients residing in Front Range communities that are more accessible to NJH. Finally, because we did not have data on body site, we cannot specifically associate these findings with pulmonary disease. While most NTM isolates are from pulmonary sites, these findings may be generalizable to all types of NTM infection because environmental exposures likely influence both pulmonary and extrapulmonary NTM infection.

## 6. Conclusions 

We found that the presence of molybdenum in the source water of Colorado watersheds is consistently associated with increased risk of NTM disease. While this study cannot establish a causal association, numerous factors bolster the validity of our findings. Our results for molybdenum are consistent with reports in the scientific literature. The connection established between molybdenum and *M. tuberculosis* offers biological plausibility and elevated blood serum concentrations of molybdenum among NTM patients offer specificity to our finding.

This study opens many new avenues of research for the NTM research community. A water sampling study in Colorado could further support these findings, as well as understanding the dose–response relationship between molybdenum and NTM growth. Importantly, the relationship between molybdenum and NTM growth in the (human) host warrants better understanding. Answering these questions will not only improve patients’ lives, but could also contribute to the development of a prevention plan based on environmental risk factors and substantially decrease the risk of exposure and ultimately disease.

## Figures and Tables

**Table 1:** Median and standard deviation (SD) values of water-quality constituents * obtained from the Water Quality Portal (WQP) used in principal component analysis (PCA). (µg/L = micrograms per liter).

Exposure Characteristics	Median ± SD (µg/L)
Aluminum	18 ± 4371.6
Arsenic	<0.5 ± 49.9
Cadmium	0.1 ± 50.6
Calcium	32,110 ± 70,745.7
Copper	1.6 ± 440.8
Iron	38 ± 26,245.6
Lead	<0.5 ± 326.4
Magnesium	6691 ± 40,822.9
Manganese	22.6 ± 7406.7
Molybdenum	4.3 ± 18.8
Nickel	1.2 ± 37.2
pH	7.93± 0.77
Potassium	1347 ± 6884.6
Selenium	0.06 ± 48.0
Sodium	6100 ± 123,203.3
Zinc	17 ± 5951.9

* The filtered (dissolved) portion of the water sample fractions were used.

**Table 2:** Model 1: Poisson regression model examining principal components and other covariates associated with NTM disease risk. Bolded estimates are statistically significant.

Characteristics	Relative Risk (95% CI) *p*-Value
Age: ≥65 years (%)	0.969 (0.059, 15.319) 0.813
Race: Non-White ^a^	**0.118** **(0.046, 0.298)** **1.57 × 10^−6^**
Drive Time (>2.0 h to NJH)	**0.634** **(0.485, 0.821)** **1.14 × 10^−5^**
Principal Component 1	**1.054** **(1.0007, 1.111)** **0.049**
Principal Component 2	1.035 (0.979, 1.094) 0.223
Principal Component 3	**1.083** **(1.009, 1.161)** **0.026**

^a^ Reference group is White Alone.

**Table 3:** Model 2: Poisson regression model examining individual metals from principal component 1 and other covariates associated with NTM disease risk. Bolded estimates are statistically significant.

Characteristics	Relative Risk (95% CI) *p*-Value
Age: ≥65 years (%)	2.16 (0.119, 37.6) 0.599
Race: Non-White ^a^	**0.076** **(0.027, 0.211)** **8.3 × 10^−7^**
Drive Time (>2.0 h to NJH)	**0.513** **(0.393, 0.663)** **5.1 × 10^−7^**
pH	0.810 (0.543, 1.22) 0.306
Arsenic (1-log unit)	0.946 (0.869, 1.03) 0.082
Calcium (1-log unit)	**1.95** **(1.36, 2.77)** **0.0003**
Magnesium (1-log unit)	**0.625** **(0.475, 0.820)** **0.0007**
Molybdenum (1-log unit)	**1.22** **(1.07, 1.39)** **0.0030**
Potassium (1-log unit)	0.988 (0.785, 1.24) 0.918
Selenium (1-log unit)	0.966 (0.906, 1.03) 0.297
Sodium (1-log unit)	1.04 (0.882, 1.23) 0.637

^a^ Reference group is White Alone.

**Table 4:** Model 3: Poisson regression models examining selected metals from Model 2 and other covariates associated with NTM disease risk in three separate single-exposure models. Bolded estimates are statistically significant.

Characteristics	Relative Risk (95% CI) *p*-Value	Characteristics	Relative Risk (95% CI) *p*-Value	Characteristics	Relative Risk (95% CI) *p*-Value
Age: ≥65 years (%)	1.32 (0.08, 20.5) 0.845	Age: ≥65 years (%)	1.42 (0.092, 21.0) 0.801	Age: ≥65 years (%)	0.86 (0.05, 13.7) 0.916
Race: Non-White^a^	**0.14** **(0.06, 0.32)** **2.4 × 10^−6^**	Race: Non-White ^a^	**0.20** **(0.09, 0.44)** **5.9 × 10^−6^**	Race: Non-White m ^a^	**0.16** **(0.07, 0.34)** **3.7 × 10^−6^**
Drive Time (>2.0 h to NJH)	**0.52** **(0.40, 0.66)** **1.7 × 10^−7^**	Drive Time (>2.0 h to NJH)	**0.55** **(0.43, 0.69)** **9.2 × 10^−7^**	Drive Time (>2.0 h to NJH)	**0.55** **(0.43, 0.69)** **7.54 × 10^−7^**
Calcium (1-log unit)	**1.19** **(1.05, 1.35)** **0.0055**	Magnesium (1-log unit)	1.06 (0.96, 1.17) 0.232	Molybdenum (1-log unit)	**1.17** **(1.05, 1.29)** **0.0041**

^a^ Reference group is White Alone.

**Table 5:** Model 4: Poisson regression model examining individual metals from principal component 3 and other covariates associated with NTM disease risk. Bolded estimates are statistically significant.

Characteristics	Relative Risk (95% CI) *p*-Value
Age: ≥65 years (%)	1.17 (0.065, 20.1) 0.915
Race: Non-White ^a^	**0.09** **(0.03, 0.26)** **1.1 × 10^−5^**
Drive Time (>2.0 h to NJH)	**0.57** **(0.44, 0.73)** **1.3 × 10^−5^**
pH	0.99 (0.68, 1.45) 0.964
Aluminum (1-log unit)	1.01 (0.96, 1.07) 0.615
Arsenic (1-log unit)	**0.90** **(0.82, 0.99)** **0.022**
Cadmium (1-log unit)	1.00 (0.96, 1.04) 0.917
Manganese (1-log unit)	1.06 (0.96, 1.18) 0.255
Molybdenum (1-log unit)	**1.23** **(1.06, 1.41)** **0.0049**
Selenium (1-log unit)	1.00 (0.94, 1.06) 0.899
Zinc (1-log unit)	1.02 (0.93, 1.13) 0.653

^a^ Reference group is White Alone.

**Table 6:** Model 5: Poisson regression models examining selected metals from Model 4 and other covariates associated with NTM disease risk in two separate single-exposure models. Bolded estimates are statistically significant.

Characteristics	Relative Risk (95% CI) *p*-Value	Characteristics	Relative Risk (95% CI) *p*-Value
Age: ≥65 years (%)	2.12 (0.14, 30.4) 0.583	Age: ≥65 years (%)	0.86 (0.051, 13.7) 0.916
Race: Non-White ^a^	**0.23** **(0.11, 0.48)** **8.3 × 10^−5^**	Race: Non-White ^a^	**0.16** **(0.07, 0.34)** **3.7 × 10^−6^**
Drive Time (>2.0 h to NJH)	**0.56** **(0.44, 0.70)** **1.4 × 10^−6^**	Drive Time (>2.0 h to NJH)	**0.55** **(0.43, 0.69)** **7.54 × 10^−7^**
Arsenic (1-log unit)	0.96 (0.89, 1.01) 0.113	Molybdenum (1-log unit)	**1.17** **(1.05, 1.29)** **0.0041**

^a^ Reference group is White Alone.

## Supplementary Materials

The following are available online at https://www.mdpi.com/1660-4601/17/11/3854/s1, Supplementary Table S1. Nontuberculous mycobacteria (NTM) species from patient isolates. Supplementary Table S2. Water-quality constituents extracted from the Water Quality Portal (WQP). Supplementary Table S3. Percent contribution of each metal and nonmetal to each principal component. Supplementary Table S4. Correlation matrix for the most highly contributing water-quality constituents to Principal Components 1 and 3. Supplementary Table S5. Sensitivity analysis examining the significant metals using negative binomial model. Our R source code is provided to calculate the watershed medians for water-quality constituents (“WatershedMedianCalcs.R”). Appendix S1. Data cleaning documentation, GIS files—watershed boundary datasets (HUC-8 and HUC-10).
