# Supplementary material for: Nontuberculous Mycobacterial Disease and Molybdenum in Colorado Watersheds
Source: Int J Environ Res Public Health. 2020 May 29;17(11):3854. doi: 10.3390/ijerph17113854 (PMC7312647; doi:10.3390/ijerph17113854)
Supplement: Supplementary file 1 [file ijerph-17-03854-s001.pdf]

Supplementary Table S1. Nontuberculous mycobacteria (NTM) species from patient isolates.
"M." is an abbreviation for "*Mycobacterium*".

**NTM species**	
M. abscessus	*M. malmoense*
M. alvei	*M. marseillense*
M. arosiense	*M. massiliense*
M. arupense	*M. mucogenicum*
M. avium	*M. nebraskense*
M. avium complex	*M. neoaurum*
M. bolletii	*M. nonchromogenicum*
M. bouchedurhonense	*M. paraffinicum*
M. brisbanense	*M. parascrofulaceum*
M. celatum	*M. peregrinum*
M. chelonae	*M. phocaicum*
M. chimaera	*M. porcinum*
M. colombiense	*M. rhodesiae*
M. conceptionense	*M. scrofulaceum*
M. flavescens	*M. senegalense*
M. fortuitum	*M. septicum*
M. gadium	*M. shimoidei*
M. genavense	*M. simiae*
M. goodii	*M. smegmatis*
M. gordonae	*M. szulgai*
M. interjectum	*M. timonense*
M. intracellulare	*M. triplex*
M. kansasii	*M. vulneris*
M. kumamotonense	*M. wolinskyi*
M. kyorinense	*M. xenopi*
M. lentiflavum	*M. yongonense*
M. mageritense	

Supplementary Table S2. Water-quality constituents extracted from the Water Quality Portal (WQP) [18]. https://www.waterqualitydata.us/portal/

**Major Metals**	**Minor Metals**	**Major Nonmetals**	**Minor Nonmetals**	**Other water-quality characteristics**
Sodium	*Aluminum*	Alkalinity	Antimony	*pH*
Potassium	Barium	Bicarbonate	*Arsenic*	Specific conductance
Magnesium	Beryllium	Bromide	Boron	Streamflow
Calcium	*Cadmium*	Carbon dioxide	*Selenium*	Total coliform
	Cerium	Carbon		
	Chromium	Carbonate		
	Chromium(III)	Chloride		
	Chromium(VI)	Fluoride		
	Cobalt	Hydrogen ion		
	Copper	Inorganic carbon		
	Dysprosium	Oxygen		
	Erbium	Silica		
	Europium	Silicon		
	Gadolinium	Sulfate		
	Iron			
	Lanthanum			
	Lead			
	Lithium			
	Manganese			
	Mercury			
	Molybdenum			
	Neodymium			
	Nickel			
	Praseodymium			
	Rhenium			
	Rubidium			
	Samarium			
	Scandium			
	Silver			
	Strontium			
	Thallium			
	Titanium			
	Vanadium			
	Ytterbium			
	Yttrium			
	Zinc			

*Italicized variables were included in principal component analysis.

Supplementary Table S3.
Percent contribution of each metal and nonmetal to each principal component.
(%, percent contribution)

**Characteristic**	**Principal Component 1 (%)**	**Principal Component 2 (%)**	**Principal Component 3 (%)**
Aluminum	0.0278	17.5	4.04
Arsenic	3.18	8.58	19.4
Cadmium	1.26	7.51	8.49
Calcium	15.9	1.52	1.86
Copper	0.861	13.9	0.660
Iron	2.97	8.62	0.010
Lead	0.526	12.0	2.16
Magnesium	17.1	0.961	0.671
Manganese	1.47	3.35	37.2
Molybdenum	9.08	0.016	4.29
Nickel	2.16	2.43	0.691
pH	6.77	4.68	6.80
Potassium	14.6	0.492	1.02
Selenium	7.37	4.04	8.40
Sodium	16.5	0.647	0.730
Zinc	0.206	13.9	3.51

Supplementary Table S4. Correlation matrix (coefficient of determination, R^2) for the contributing water-quality constituents to Principal Components 1 & 3.

	Al	As	Cd	Ca	Mg	Mn	Mo	pH	K	Se	Na	Zn
Aluminum (Al)	1.000											
Arsenic (As)	0.55	1.00										
Cadmium (Cd)	0.32	0.22	1.00									
Calcium (Ca)	-0.25	0.14	0.09	1.00								
Magnesium (Mg)	-0.20	0.22	0.10	0.95	1.00							
Manganese (Mn)	0.15	0.06	0.31	0.26	0.25	1.00						
Molybdenum (Mo)	0.08	0.34	0.14	0.46	0.50	-0.01	1.00					
pH	-0.28	0.13	-0.19	0.57	0.58	-0.18	0.34	1.00				
Potassium (K)	-0.16	0.25	0.20	0.78	0.79	0.27	0.49	0.50	1.00			
Selenium (Se)	0.31	0.57	0.18	0.39	0.46	0.12	0.52	0.14	0.33	1.00		
Sodium (Na)	-0.17	0.21	0.16	0.83	0.88	0.27	0.55	0.55	0.84	0.45	1.00	
Zinc (Zn)	0.53	0.32	0.44	-0.04	-0.03	0.38	0.17	-0.25	-0.08	0.29	-0.05	1.00

Supplementary Table S5.

Sensitivity analysis examining the significant metals from Model 3 using negative binomial model[a] ($p<0.05$).

**Characteristics**	Relative Risk 95% CI (p-value)	**Characteristics**	Relative Risk 95% CI (p-value)
Molybdenum (1-log unit)	**1.28 1.04, 1.58 (0.024)**	Calcium (1-log unit)	**1.28 1.03, 1.61 (0.028)**

[a]Each model is also controlled for age, race, and drive time.
